# Application and progress of new technologies and new materials in the treatment of pathological scar

**DOI:** 10.3389/fchem.2024.1389399

**Published:** 2024-05-01

**Authors:** Yining Liu, Sisi Wang, Fan Yang, Xuepeng Wang, Jierui Zhang, Xinkun Han, Xipeng Zhang, Zhiguo Wang

**Affiliations:** ^1^ The Affiliated Hospital of Qingdao University, Qingdao University, Qingdao, China; ^2^ Qingdao Municipal Hospital, University of Health and Rehabilitation Sciences (Qingdao Municipal Hospital), Qingdao, China; ^3^ Department of Burn and Plastic Surgery, the Affiliated Hospital of Qingdao University, Qingdao, China

**Keywords:** pathological scar, new technologies, new biological materials, microneedle (MN), photodynamic therapy (PDT), photosensitizer, exosome (EXO)

## Abstract

Pathological scars (PS), including hypertrophic scars (HTS) and keloids, are a common complication of poor wound healing that significantly affects patients’ quality of life. Currently, there are several treatment options for PS, including surgery, drug therapy, radiation therapy, and biological therapy. However, these treatments still face major challenges such as low efficacy, high side effects, and a high risk of recurrence. Therefore, the search for safer and more effective treatments is particularly urgent. New materials often have less immune rejection, good histocompatibility, and can reduce secondary damage during treatment. New technology can also reduce the side effects of traditional treatments and the recurrence rate after treatment. Furthermore, derivative products of new materials and biomaterials can improve the therapeutic effect of new technologies on PS. Therefore, new technologies and innovative materials are considered better options for enhancing PS. This review concentrates on the use of two emerging technologies, microneedle (MN) and photodynamic therapy (PDT), and two novel materials, photosensitizers and exosomes (Exos), in the treatment of PS.

## 1 Introduction

Scar formation results from abnormal chronic inflammation of the wound area, which is characterized by excessive proliferation of fibroblasts and abnormal deposition of collagen (Tosa et al., 2016). Moreover, various cellular factors such as growth factors, cytokines and chemokines are involved in the scarring process. Besides, the formation of scars is influenced by an individual’s race, gender, and age, as well as the tension, location, and mode of injury of the wound (Blakytny and Jude, 2006). Currently, keloid and HTS can be treated with surgery, local corticosteroid injection, and physical therapy. However, these monotherapies are associated with a high rate of recurrence.

In response to the limitations of scar treatment, researchers have been extensively investigating a variety of new therapeutic techniques and materials for PS. One such technology is microneedles, which have been applied in various forms due to their efficient subcutaneous delivery of drugs. MNs are a minimally invasive and painless way to continuously release drugs, which can help avoid the side effects of intravenous and oral drugs on patients. There are several main forms of MNs, including solid, coated, hollow, soluble, and hydrogel-based MNs (Xu et al., 2021). When delivering drugs to the skin, microneedle systems often use novel materials as drug carriers to improve the drug utilization in scar tissue. In addition to MNs, PDT is considered as a promising new technique for the treatment of PS because of its temporal and spatial accuracy and less invasiveness (Wen et al., 2017). The efficacy of this therapy depends, to some extent, on the accumulation of intracellular photosensitizers. Therefore, improving the performance of photosensitizers is crucial for enhancing the therapeutic effect of PDT on pathological scars. To achieve this, scholars are exploring the use of nanoparticles made of new materials to enhance the efficacy of photosensitizers in PDT. Consequently, new material-supported photosensitizers have emerged as a significant area of research in optical therapy (Escudero et al., 2021). New materials can serve as carriers for targeted drugs or photosensitizers to assist new therapeutic technologies. Additionally, some biological materials can have a therapeutic effect on pathological scars. For instance, exosomes are nanoscale vesicles secreted by mesenchymal stem cells (MSCs) that contain genetic material corresponding to MSCs and a large number of miRNAs. Therefore, exosomes can perform similar functions to parental cells and act as mediators in cell communication, transmitting cell signals (Horibe et al., 2018; Mathieu et al., 2019). In addition, exosomes can exist stably *in vivo* for a long time without immune rejection and can improve drug activity. This makes them a promising biological material for drug delivery (Rajput et al., 2022). To sum up, in addition to using new technologies and new materials separately, scholars have also combined them in various ways to further expand their benefits and improve the effect of scar treatment.

**FIGURE 1:**
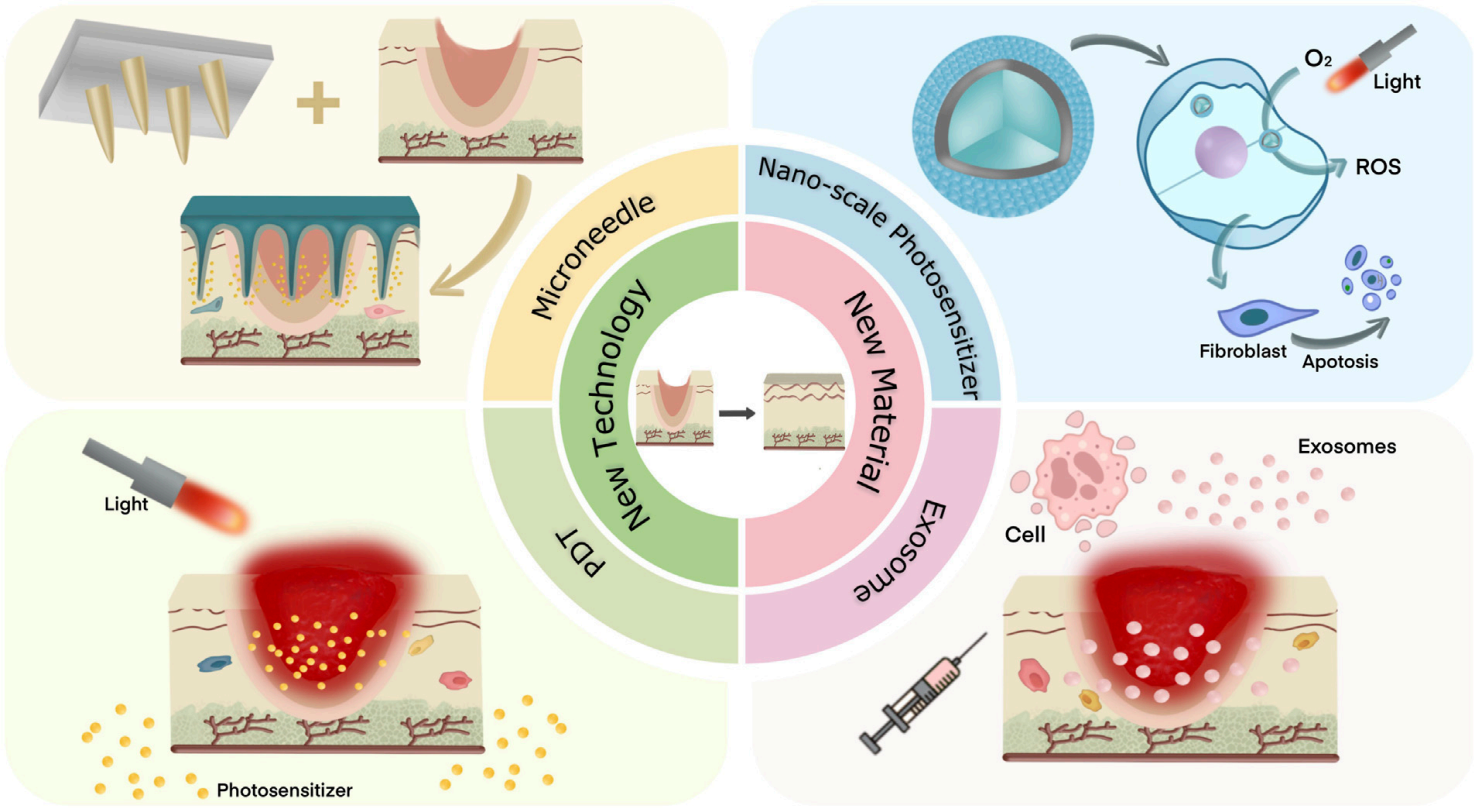
Two new techniques and two new materials for the treatment of pathological scar were introduced.

This review presents some new techniques and materials for the treatment of PS that have therapeutic effects (See Figure 1). The specific application forms of these technologies and materials are listed, and the design principles of derivative products of different materials are analyzed. The related mechanisms of these technologies and materials in inhibiting hyperplasia of scars are explored, and the prospect of joint application of new technologies and new materials is also discussed.

## 2 New technologies

### 2.1 Microneedle (MN)

Microneedle (MN) is a minimally invasive transdermal drug delivery system that enables simple, efficient and painless administration (Wang et al., 2017). Its main forms are solid MNs, coated MNs, hollow MNs, soluble MNs, and hydrogel-based MNs. MNs deliver therapeutic substances such as small molecules, biological macromolecules and exosomes to the local skin by forming tiny pores in the stratum corneum (Lin et al., 2019; Luo et al., 2019; Yang et al., 2019). This method of painless delivery of drugs is more acceptable to patients than the traditional method of intravenous administration. In contrast to oral drugs, transdermal delivery of drugs does not go through the gastrointestinal tract and directly to the site of the affected skin, so the risk of systemic side effects is reduced (Morry et al., 2015). Not only that, MNs can also improve drug utilisation. For example, the swellable needle tips made by Shi et al. (2022) to improve hair loss were able to greatly extend the release cycle of hair growth activators. This sustained release avoids the metabolism or degradation of the drug that would result from early mass release and improves the utilisation cycle of the drug. Similarly, MNs with a heterogeneous matrix (Shi et al., 2024) are able to constrain drug within the needles and avoid drug diffusion into the substrate, which result into low-efficient drug usage.

Based on these therapeutic advantages, new technologies such as MNs have been widely utilized in clinical medicine. The therapeutic principles of MN systems for PS focus on both anti-inflammatory and antifibrotic aspects. According to the therapeutic principles, the materials used to make MNs are not only biocompatible and have good biodegradability, but also have curative effects and are able to synergize with drugs to achieve a better therapeutic effect. Therefore, a complete MN system usually consists of a MN patch with good biocompatibility, biodegradability and mechanical strength, as well as a drug that is compatible with the therapeutic principle. Due to the low solubility of some drugs, it is difficult to reach the treatment sites. Therefore, in order to improve the utilization of drugs, it is necessary to choose a suitable carrier to encapsulate the drugs when using MNs for transdermal drug delivery, and then mix the drug-carrying particles with MN patches. In recent years, nanoparticles made of various new materials have been considered as good materials for loading drugs because of their superior properties. Jiang et al. (2022) believe that nanoparticles can increase the contact area between drugs and action sites, improve the dissolution rate of drugs, as well as increase the bioavailability of drugs. As a drug carrier, nanoparticles have a very high drug loading capacity, and the non-polymer covered structure makes the drug loading capacity of nanoparticles even reach 100%. On top of that, the nanoscale structure dissolves slowly in a liquid environment and can release drugs at the treatment site for a long time. When combined with the MNs, the drug-carrying nanoparticles can be delivered deep into the skin, avoiding the difficulty of reaching the treatment site due to the drug remaining above the stratum corneum. Therefore, in order to improve the therapeutic effect, most MN systems have drug-carrying nanoparticles made of different materials within them (See Table 1).

**TABLE 1:** Summarizing of application of different microneedles in the treatment of pathological scar.

Name	Type	Material	Advantage	Disadvantage	Therapeutic principle
5-FU-CMC-MN Park and Kim (2021)	Coated MN	5-FU, CMC	Hydrophilia, biodegradability, antimicrobial activity, wound healing properties, low surface tension, local drug release	Low drug loading	5-FU-CMC affects DNA synthesis and collagen expression associated with scar formation by inhibiting TGF-β1
BSP-MNs-QUE@HSF/CDF Wu et al. (2021)	Dissolvable MN	BSP, CDF, QUE, HSFs membrane	High drug loading, biocompatibility, wound healing properties, promote coagulation, anti-inflammatory, antioxidant activities, high mechanical strength, homologous targeting	The specific proteins related to the homologous targeting function of HSFs are still unknown	Reducing the expression of collagen I and collagen III in hypertrophic scar by regulating Wnt/β-catenin and JAK2/STAT3 pathways
UCNPs@mSiO2-HA-MNs Wang et al. (2020)	Dissolvable MN	HA, UCNP, mSiO2, siRNA of TGF-βR1	Protect siRNA from degradation, gene delivery, gene monitoring, gene regulation, dynamic observation	Unsustainably released	siRNA of TGF-βR1 can suppress the over-expression of mRNA of CTGF in hypertrophic scar and inhibit the overgrowth of fibroblasts and the overproduction of collagen
MOFs (CuOx@MIL-101)-CQ-MNs Chen et al. (2023)	Dissolvable MN/light-driven MN	MOFs,CQ, starch, gelatin	Exceptional stability, ample space, a high yield of hydroxyl radicals, good mechanical strength, improve transdermal transmission of photosensitizers	Not mentioned	Hydroxyl radicals leads to cell death and decreased collagen deposition. CQ interfere with the protective autophagy of cells. LC3II/I ratio and the expression of COL-1 and TGF-β1 is decreased, and the expression of P62 is increased
GelMA/AgNC/TEG/ZIF-8-MNs Zhao et al. (2023)	Dissolvable MN	GelMA, AgNCs, TRG, ZIF-8	Displaystunable mechanical property, biocompatible, biodegradable, lower fabrication cost, high production yield, ipH-responsivity, antibacterial activity for infection prevention, inhibit the major regulatory pathway of ferroptosis	Not mentioned	Promoting ferroptosis in myofibroblasts, further achieving the purpose of anti-fibrosis

As you can see, choosing the right fabrication materials is the key to improving the effectiveness of MN treatments. For exampe, Both 5-fluorouracil carboxymethyl chitosan nanoparticle microneedles (5-FU-CMC-MNs) designed by Park and Kim (2021) and an active targeting drug delivery system (BSP-MNs-QUE@HSF/CDF) designed by Wu et al. (2021) combine antibacterial and anti-inflammatory biomaterials with anti-fibrotic therapeutic agents. Park’s choice of CMC nanoparticles are antibacterial, anti-inflammatory and promote wound healing. It carries 5-FU, which has anti-metabolic activity and can suppress the synthesis of deoxyribonucleic acids, leading to apoptosis of the pathological cells. Both CMC and 5-FU can inhibit the expression of TGFβ1, a key protein in the process of scar formation. Thus, 5-FU-CMC affects DNA synthesis and collagen expression associated with scar formation by inhibiting TGF-β1 and further inhibits the proliferation of keloid fibroblasts (KFs). Similarly, Bletilla striata polysaccharide (BSP) are also able to accelerate wound healing, promote coagulation, anti-inflammatory and anti-oxidation. Wu utilized BSP to create soluble MN patches, and delivered quercetin (QUE) into HTS to regulate Wnt/β-catenin and JAK2/STAT3 pathways and to decrease the expression of collagen I and collagen III. Among them, the cyclodextrin metal-organic framework (CDF), as the carrier of anti-fibrosis drugs, was coated with hypertrophic scar fibroblast (HSF) membrane to form drug-carrying nanoparticles (QUE@HSF/CDF). In addition to enhancing drug permeability, most importantly, QUE@HSF/CDF can take advantage of the homologous targeting effect of HSFs and utilize the complex properties of the cell membranes to disguise itself as a homologous substance, so that drugs can actively target HSFs. Hence, this active targeted drug delivery method not only avoids the skin retention of the drug carrier due to lack of specific adhesion, but also greatly reduces the drug depletion during transdermal delivery.

In addition to delivering drugs, MNs are also capable of delivering siRNA with anti-fibrotic properties to treat PS and monitor the extent of treatment. For example, the UCNPs@mSiO2-HA microneedle system produced by Wang et al. (2020) supresses the overexpression of connective tissue growth factor (CTGF) mRNA in HTS by dissolving and delivering siRNA of TGF-βR1, thereby inhibiting the excessive proliferation of fibroblasts and the overproduction of collagen. With unique optical properties, UCNPs, the carrier of siRNA, can track the dynamic insertion process through OCT imaging and detect the insertion depth via the red-blue signal ratio. To observe the therapeutic effect of siRNA, molecular beacons (MB) of CTGF were introduced into UCNPs to enable gene monitoring of therapeutic siRNA. Based on this, the MN patch can dynamically observe the transdermal condition of MN and monitor the therapeutic effect.

Besides the anti-inflammatory and antifibrotic mentioned above, in recent years, initiating ferroptosis in myofibroblasts is a new strategy for the treatment of HTS (Lei et al., 2022). Ferroptosis is a type of non-apoptotic cell death characterized by loss of glutathione (GSH), reduced glutathione peroxidase 4 (GPX4) activity, and excessive production of reactive oxygen species (ROS). The depletion of GSH is the main cause of ferroptosis (Li et al., 2023). Studies have shown that myofibroblasts in HTS exhibit high iron levels, which can be targeted for “Ferroptosis-mediated scarring therapy.” To this end, Zhao et al. (2023) developed a new ferroptosis-based nanoplatform by combining AgNC/TRG/ZIF-8 with the GelMA MN patch. In acidic environment, ZIF-8 is easy to degrade and release Zn2+ and 2-methylimidazole ligand. Zn2 + can consume the intracellular GSH, and trigger the generation of ROS in the cell. Simultaneously, 2-methylimidazole ligands can enhance phagocytosis through strong electrostatic attraction. Silver nanoclusters (AgNCs) exhibit potent antibacterial activity for infection prevention and can also act as active nanoagents by consuming GSH. Trigonelline (TRG), a Chinese herbal medicine, significantly inhibits the nuclear factor erythroid 2-related factor 2 (NRF2) pathway, which is the main regulatory pathway of ferroptosis. More importantly, the three components released by the composite synergistically promote the accumulation of lipid peroxidation and reduce GPX4 activity. Therefore, the nano-sized AgNC/TRG/ZIF-8 composite promotes ferroptosis in myofibroblasts in a highly synergistic manner after degradation in acidic lysosome, further achieving the purpose of curing HTS.

In addition to the single use of MNs, the combination of photodynamic therapy (PDT) and MNs has begun to receive widespread attention in recent years. This therapeutic method utilizes photothermal effect and transdermal drug delivery for the treatment of PS. This combination of multiple new technologies is centered on light-driven MNs with therapeutic photosensitizing drugs within them. This therapeutic principle behind this combination of multiple new technologies is to use light to drive a therapeutic photosensitive drug within the MNs. For instance, Chen et al. (2023) designed a light-driven MN patch to deliver the photosensitizer MOFs (CuOx@MIL-101) and the autophagy inhibitor chloroquine (CQ) deep into the HTS. It is important to note that Metal-organic frameworks (MOFs) can generate a high level of hydroxyl radicals when exposed to visible light, which can cause cell death and reduce collagen deposition. Additionally, CQ can hinder the protective autophagy of cells by preventing autophagosomes from fusing with lysosomes, leading to the accumulation of mature but ineffective autophagic vacuoles. Therefore, the combined treatment augmented the toxicity of HSFs. It also downregulated the expression of collagen type I and TGF-β1, decreased the LC3II/I ratio, and increased the expression of P62, which is a marker of autophagic flux. Consequently, this led to the demise of HSFs.

In summary, the MN transdermal drug delivery system has now built a bridge between drugs and the human body. Various forms of MNs have gradually become a new way to prevent and treat PS. And the combination therapy derived from MNs and other technologies has also expanded its application in other fields such as biomedicine and clinical.

### 2.2 Photodynamic therapy (PDT)

**FIGURE 2:**
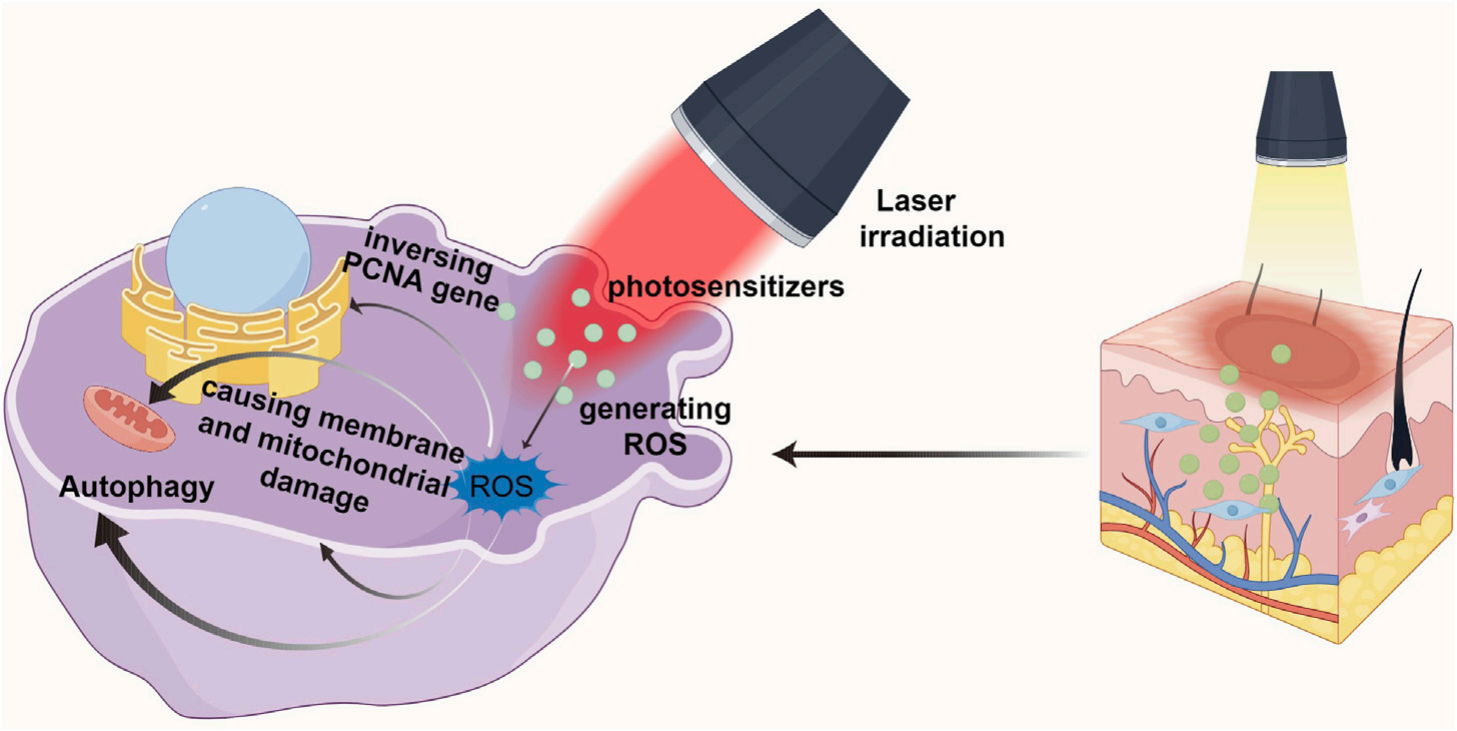
The process and principle of photodynamic therapy in the treatment of pathological scars.

Photodynamic therapy (PDT) is an effective local treatment for malignant skin diseases and many other types of tumors. PDT consists of three basic elements: photosensitizer, laser irradiation, and production of reactive oxygen species (ROS). Its efficacy depends in part on the accumulation of intracellular photosensitizers (Ritz et al., 2012). PDT embodies the principle of non-invasive and safe treatment: laser is used to activate the photosensitizer applied in the diseased tissue, so that it interacts with molecular oxygen in the surrounding tissue, producing cytotoxic ROS and free radicals to selectively destroy the normal physiological state of the relevant cells (Tosa and Ogawa, 2020) (See Figure 2).

In recent years, PDT has been recognized as a promising approach for the treatment of PS due to its great spatio-temporal accuracy and minimal invasibility (Wen et al., 2017). The mechanism is to inhibit HSF proliferation and induce HSF apoptosis through ROS (Jiang et al., 2019; Li et al., 2019). Tosa and Ogawa (2020) mentioned that after scar tissue underwent PDT, PCNA gene expression related to fibroblast proliferation showed an inverse pattern. Moreover, PDT decreased the expression of type I and III collagen genes in scar tissue, increased the expression of MMP-3 and proelastin as well as improved the matrix composition. Studies have shown that KFs can be killed after being treated with high energy 5-ALA-PDT. Under lower energy laser irradiation, the fibroblasts could survive, but the contraction ability and collagen production decreased significantly. In addition, the combination of different photosensitizers and laser intensity has a great influence on the effect of PDT on the inhibition of fibroblasts.

Heckenkamp et al. (2004) found that ROS in can cause membrane and mitochondrial damage, and then activating signal molecules and changing the expression of growth factors and cytokines in scar tissue, thus regulating the production and distribution of collagen and extracellular matrix. Not only that, they also found that PDT reduced the proliferation and migration of normal dermal fibroblasts grown in the gel matrix, and significantly limited the expression of TGF-β1 and basic fibroblast growth factor mRNA. In addition, previous studies believed that fibroblast death caused by PDT was mostly a process of apoptosis and necrosis (Hu et al., 2017). However, Liu et al. (2019) found that the principle of 5-ALA-PDT was not through inducing apoptosis as emphasized in previous studies. Instead, it reduces the incidence of keloid by promoting autophagy of keloid fibroblasts. That is, PDT may lead to superoxide anion dependent autophagy death. Similarly, Niu et al. (2021) also demonstrated the relationship between PDT and autophagy. The natural photosensitizer hypocrellin A (HA) combined with red laser irradiation (HA-R-PDT) inhibited the TGF-β/Smad signaling pathway in KFs. TGF-β1 promotes in KFs through Smad and ERK pathways, while inhibition of autophagy changes TGF-β1 expression through negative feedback. Thus, HA-R-PDT inhibits KFs cell hyperproliferation, collagen synthesis and ECM accumulation by regulating TGF-β1-ERK-autophagy apoptosis signaling pathway.

Although PDT can provide adjuvant treatment to the wound after scar resection and prevent the recurrence of keloid after surgery. However, PDT is a relatively superficial treatment due to the limited penetration of photosensitizers and lasers. Although the fibrosis of scar can be reduced after multiple PDT, the current photosensitizer and photoconduction technology are difficult to ensure the depth of treatment and rapid PDT response. Therefore, the development of photosensitizers is a key influencing factor to improve the efficiency of PDT.

## 3 New materials

### 3.1 New photosensitizer

PDT is a new therapeutic strategy for HTS. Since the therapeutic effect of PDT is closely related to photosensitizer, the improvement of photosensitizer properties will greatly improve the efficacy of PDT. In addition to traditional chemical agents, new photosensitizers made through nanotechnology and new materials are also able to respond to external photothermal stimuli. For example, aggregation-induced emission (AIE), also known as AIE luminescent materials or AIEgens, emit enhanced light when aggregating. AIE can aviod fluorescence attenuation or quenching in concentrated solution or aggregation, insufficient production of reactive oxygen species, which can enhance PDT effect (Kwok et al., 2015). In addition, the nanostructures accumulate in tumors and damaged tissues by their permeability and retention effects (EPR). This passive targeting helps to target the nanostructures photosensitizers into the treatment area of PDT.

Some nanoparticles (NPs) can not only be used as photosensitizers or active oxygen sources, but also carry photosensitizers as functional carriers. The nanostructured molecular counterpart has higher chemical stability, and can also be used as a quenching agent for the emission of active photosensitizer, which improves the production efficiency of ROS. NPs combined with novel materials provide a wider range of excitation possibilities in PDT, such as core-shell nanostructures of titanium dioxide coated conversion nanoparticles (UCNPs) capable of absorbing near-infrared radiation and emitting visible and ultraviolet light. Other nanostructures of different materials can be combined with the photosensitizer or incorporated into the porous structure, such as inorganic nanoparticles which include gold nanoparticles, silica and its derivatives, iron oxide nanoparticles (iONPs) and up-conversion nanoparticles (UCNPs); Micelles and vesicles; Liposome; The porous support including mesoporous silica and metal-organic skeleton (MOFs) (Escudero et al., 2021). Most photosensitizers have poor stability and solubility, incorporating them into nanostructured carriers improves delivery efficiency during PDT, and can also respond to specific stimuli and allow for spatiotemporal controlled release of drugs (Couvreur et al., 2006; Mura et al., 2013).

Most photosensitizers are hydrophobic, so the hydrophobic core of micelles makes them ideal carriers for PDT (Xue et al., 2019). In order to achieve the high efficiency and multiplicity of PDT, compared with micelles, liposomes are not only very suitable for the inclusion of hydrophobic drugs, but also can incorporate hydrophilic and hydrophobic drugs at the same time, which makes it possible to design multiple treatment systems (Song et al., 2017). Moreover, liposomes can control cargo release and reduce its clearance rate. Liposomes also direct themselves into a target site in the human body, reducing the side effects of PDT such as cytotoxicity (Gross et al., 2013). Nanoscale metal-organic frameworks (NMOFs) show obvious advantages in the PDT process. For example, the special structure of NMOFs enables them to have a significantly high ratio surface area, allowing binding numerous photosensitizers. Its clear porosity allows the photosensitizers to be isolated from each other, avoiding photosensitizers’s self-quenching, and improving the therapeutic efficiency of PDT. Endogenous degraded NMOFs can be activated by light, providing a new platform for the application of activated nanoscale photosensitizers in PDT.

In recent years, the combination mode of biomaterial ethosomes (ESs) and photosensitizers has achieved remarkable effect in PDT treatment of pathological scars (See Table 2).

**TABLE 2:** Summarizing of different nano-photosensitizers for PDT in the treatment of PS.

Name	Material	Characteristics
IR-808-ES Yu et al. (2021)	IR-808, ethosomes	(1) Exhibit aggregation induced emission enhancement (AIEE) phenomenon
		(2) Induce HSF apoptosis through intrinsic mitochondrial pathway
		(3) Strong tissue penetration
A/A-ES Chen et al. (2021)	ANCs, ethosomes	(1) Good coding delivery capability and catalase activity
		(2) Automatically generate O2, increase the production of ROS and improve the anoxic microenvironment
HA/ES-ALA Chen et al. (2022)	HA, ANCs, ethosomes	(1) Significant transdermal transmission
		(2) Actively target fibroblasts with HA receptors
		(3) High drug loading and release amount

As an ideal photosensitizer for PDT and photothermal therapy (PTT), IR-808 can be combined with ESs to prepare a new nanophotosensitizer for the treatment of HTS: IR-808-ES (Yu et al., 2021). The penetration depth of IR-808 near infrared excitation wavelength (≈800 nm) can reach the deep dermis. IR-808 is hydrophobic, while ES has the characteristics of passive or actively loaded hydrophobic photosensitizer. IR-808 is also fat-soluble, it forms an aggregation state after binding with ES. Depending on the aggregation environment provided by the lipid bilayer, IR-808 does not undergo fluorescence quenching for PDT, but exhibits aggregation induced emission enhancement (AIEE) phenomenon. AIEE enhances ROS production and photothermal effect.

PDT and PTT synergistic therapy (PDT/PTT effect) combined with IR-808-ES can greatly improve the effect of HTS therapy. PTT can not only convert the light energy absorbed by the photosensitizer into heat energy, but also improve the cellular uptake rate of the photosensitizer and enhance the generation of ROS. This novel synergistic therapy can cauterize the target cells, and improve the therapeutic effect of PDT. In other words, the PDT/PTT effect combines the ROS generating power of PDT with the thermal therapeutic effect of PTT.

Yu et al. (2021) showed that IR-808-ES is a new photosensitizer, and the ES in its structure can enhance the tissue penetration of IR-808 under near-infrared light irradiation. Moreover, the transdermal delivery of ES can enhance the synergistic treatment effect of PDT/PTT on HTS. *In vitro* experiments have shown that the structure of ES contributes to the accumulation of IR-808 in HSF and induces HSF apoptosis through intrinsic mitochondrial pathway. *In vivo* transdermal studies have shown that ES promotes IR-808 to penetrate the collagen fibers of HTS tissue into the deep dermis, ensures that IR-808 is delivered to HSF in a complete structure. In addition, IR-808-ES in PDT/PTT can reduce scar thickness, improve collagen deposition, and promote the recovery of HTS.

Besides IR-808-ES, A/A-ES, composed of ALA and ultra-small Au nanoclusters (ANCs), is also one kind of new nanoscale photosensitizers using ES as carriers (Chen et al., 2021).

As a conventional photosensitizer, 5-aminolevulinic acid (ALA) can bind to ES (A/A-ES) and improve the effect of ALA in PDT through transdermal administration. Based on this, the bovine serum albumin (BSA) in ANCs can fix ANCs in the lipid bilayer membrane of ES by electrostatic action, so that it exists on the surface of A/A-ES. In A/A-ES, ALA is in the core and ANCs is on the surface. The two play their respective advantages to form a functional transdermal ES. This unique structure has good coding delivery capability and catalase activity, and deliver ALA and ANCs into HTS tissue together.

Poor vascularization of HTS can easily result in a hypoxia microenvironment, causing insufficient ROS production. The reduction of ROS will reduce the effect of HTS-PDT. Therefore, compared with single ALA-ES, the addition of ANCs can automatically generate O2, increase the production of ROS, improve the hypoxia microenvironment. A/A-ES have a more obvious killing effect on cells and induce more apoptosis of HSF.

A/A-ES not only have the characteristics of transdermal co-transmission, but also enhance the efficacy of PDT by improving the color and texture of HTS tissue, inhibiting HSF proliferation, promoting HSF apoptosis, and reshaping collagen rearrangement. Based on these, A/A-ES provide an effective way to combine transdermal drug delivery with nano-enzyme in PDT.

In order to further enhance the production of ROS, Chen et al. (2022) combined A/A-ES with plasmonic resonance (LSPR) effect in cytoplasm: A/A-ES increased the production of ROS through local surface LSPR, promoted apoptosis of fibroblasts and collagen remodeling, and thus achieved the purpose of treating HTS.

ALA-ES was deformed and fractured when penetrating into HTS. During this progress, its conveying capacity was lost. Moreover, ruptured ALA-ES could not actively target HSF, limiting transmembrane transmission. To overcome the defects of ALA-ES, it was embedded in the grid of hyaluronic acid (HA). HA/ES-ALA can actively target HSF and HA receptors.

Chen et al. ’s *in vivo* and *in vitro* experiments showed that HA/ES-ALA has significant transdermal transmission ability due to its penetrating channels and membrane fusion mechanism. HA can promote transdermal delivery, and actively target fibroblasts with HA receptors. Besides, its drug loading and release amount are high. By taking advantage of the active aggregation of HA receptors on the surface of HSF, ES-ALA releases ALA from vesicles and delivers ALA to HSF through membrane fusion, significantly improving the utilization rate of ALA in HTS-PDT.

Although the combination of new materials and photosensitizers can improve the therapeutic effect of HTS-PDT, it still has obvious defects after combining with photosensitizers due to the self-limitation of biomaterials. For example, the production cost of liposome is high, the encapsulation efficiency is low, and the plasma half-life is short (Escudero et al., 2021). However, nanoparticles made of novel materials have advantages in terms of biocompatibility and biodegradability. Therefore, new photosensitizers have great potential in PDT or PTT for the treatment of PS and other diseases.

### 3.2 Exosome (Exo)

Exosomes is a kind of nanoscale extracellular vesicles containing bioactive molecules such as RNA and proteins, which can be secreted and absorbed by various types of cells. It contains specific substances from parental cells, and as a major mediator of intercellular communication. Exosomes are rich in a variety of miRNAs that regulate the function and activity of target cells and organs, and can also deliver goods to the cytosol of recipient cells through non-specific pathways such as macropinocytosis or micropinocytosis or through specific receptor-mediated processes (Mathieu et al., 2019). Therefore, in addition to expressing the characteristics of parental cells, exosomes can also act as nanoscale drug carriers for targeted drug delivery to recipient cells or deliver small molecules to recipient cells.

Based on the fact that exosomes can express similar functional properties to parental cells, and can make up for some defects of parental cells when they play a role *in vivo*, the assumption that they can replace parental cells for clinical treatment has been achieved (See Table 3). For example, adipose stem cells (ADSCs) can reduce inflammation, stimulate angiogenesis, promote wound healing, and inhibit scarring (Guisantes et al., 2012; Zhang et al., 2015; Zarei and Abbaszadeh, 2018). However, stem cell therapy can lead to the underlying tumor exhibit pathological features and non-specific differentiation. In contrast, exosomes (ADMSC-Exos) secreted by adipose mesenchymal stem cells (ADMSCs) via paracrine pathway have similar functions to ADMSCs, but do not have the side effects of ADMSCs. ADMSC-Exos has been shown to promote wound healing and regeneration (Li J. et al., 2022). Therefore, the application of exosomes in the treatment of PS has caused extensive research and exploration.

**TABLE 3:** Summarizing of different exosomes in the treatment of pathological scar.

Types of exosomes	Therapeutic principle
ADSC-Exos	Influence IL-17RA/Smad axis: (1) Regulate the inflammatory response and the proportion of Th17/Treg cells to reduce the deposition of ECM, and inhibit the proliferation and migration of fibroblasts in the later stage of wound healing
	(2) miR-192-5P in ADMSC-Exos inhibits the expression of pro-fibrotic proteins levels in HSF by targeting IL-17RA
	Promote the balance of ECM ratio or targeting collagen production: (1) Inhibite TGF-β2/Smad3 and Notch-1 signaling pathways
	(2) Inhibite MMP-1 gene expression and reduce matrix degradation necessary for cell migration
	The concentration of ADMSCs-Exos affect collagen formation and fibroblast proliferation and migration
HBMSCs-Exos	TSG-6 secreted by HBMSCs-Exos can reduce inflammatory response and collagen deposition

A large number of studies have shown that ADSC-Exos and ADMSC-Exos often avoid the formation of PS by reducing inflammation, inhibiting fibrosis and promoting the balance of ECM ratio. It is well known that TGF-β plays a key role in tissue fibrosis, affecting the transformation of fibroblasts into myofibroblasts and their proliferation, migration and secretion (Dobaczewski et al., 2011). The Smad signaling pathway is involved in almost all fibrotic diseases. TGF-β can activate Smad2 and Smad3 in the SMAD signaling pathway, thus promoting the development of fibrosis. IL-17A, as a pro-inflammatory cytokine secreted by Th17 cells, is significantly increased in HTS. The IL-17A/IL-17RA axis, in which IL-17A and its receptor IL-17RA participate, can produce TGF-β. Blocking IL-17RA, the receptor for IL-17A, can inhibit the formation of fibrosis (Meng et al., 2012; Yanagisawa et al., 2017). Therefore, the IL-17RA/Smad axis becomes the target axis for the treatment of PS.

Overproduction of inflammatory factors and persistent inflammation of the wound promote HTS formation. Th17 and Treg cells, as two types of cells that differentiate after activation of CD4+T cells, exhibit opposite roles in inflammatory response. Th17 secretes IL-17a pro-inflammatory cytokine, while Treg cells produce IL-10 anti-inflammatory cytokine. The balance between them is controlled by the inflammatory environment and affects the fibrosis of tissues and cells. ADSC-Exos has the ability to inhibit the proliferation and differentiation of T cells, reduce the release of pro-inflammatory cytokines, and coordinate the balance among various subsets of CD4+T cells (Blazquez et al., 2014; Bolandi et al., 2020). Wang et al. (2022) proved *in vivo* that ADSC-Exos could reduce the deposition of ECM by regulating the inflammatory response and the proportion of Th17/Treg cells, inhibit the proliferation and migration of fibroblasts in the later stage of wound healing, and thus reduce the generation of HTS.

In addition to influencing the anti-inflammatory effect of IL-17RA/Smad axis, TSG-6 secreted by mesenchymal stem cells (MSCs) and fibroblasts can mediate the anti-inflammatory effect of MSCs under inflammatory conditions (Hu et al., 2018). Jiang et al. (2020) have shown that TSG-6 is a potential molecular target of MSCS-derived exosomes for scar treatment, and it inhibits the formation of PS by reducing inflammatory response and collagen deposition during wound healing. Taking bone marrow mesenchymal stem cells (HBMSCs) as an example, compared with HBMSC-Exos alone, TSG-6-modified HBMSC-Exos significantly reduced inflammatory factors in PS such as MCP-1, TNF-a, IL-1b, IL-6, and related factors overexpressed during PS formation such as TGF-β1, col-1, COL-III, a-SMA, P-Smad2Ser467 and P-Smad3S423/S425. At the same time, the presence of MSCs exosomes increase the expression of TSG-6 and promote its anti-inflammatory effect in the process of scar formation.

miRNAs play a crucial role in improving skin fibrosis and promoting tissue regeneration, while exosomes regulate biological processes and intercellular communication through miRNAs (Deng et al., 2019). Li et al. (2021) found that IL-17RA was the direct target of miR-192-5P in ADMSC-Exos. ADMSC-Exos regulates the Smad pathway by interfering with IL-17RA and inhibits the expression of pro-fibrotic proteins levels in HSF, thus effectively inhibiting excessive fibrosis during wound healing. ADMSC-Exos inhibits collagen deposition and fibroblast to myofibroblast transformation via the miR-192-5P/IL-17RA/Smad axis, thereby preventing the formation of HTS.

The existing view is that promoting the balance of ECM ratio or targeting collagen production may be a new direction for the treatment of keloid. Li J. et al. (2022) found that ADMSC-Exos had an effect on ECM remodeling in keloids. ADMSC-Exos may inhibit the expression of ECM-related genes and proteins by inhibiting TGF-β2/Smad3 and Notch-1 signaling pathways, thereby reducing collagen deposition and playing an anti-fibrosis role. In addition, ADMSC-Exos can also destroy the structure of microvessels in KFs. Among them, the decrease of CD31 and CD34 positive microvessels enhanced the inhibitory effect of ADMSC-Exos on KFs. Due to enhanced TIMP-1 expression mediated by ADMSC, ADMSC-EXOS significantly inhibited MMP-1 gene expression and reduced matrix degradation necessary for cell migration (Zhou et al., 2020).

Similarly, Li C. et al. (2022) proved that high concentration of ADMSC-Exos reduced collagen formation but made collagen arrangement more orderly. Besides, high concentration of ADMSC-Exos promoted the migration and proliferation of fibroblasts, making wound healing sound. The total amount of ADMSC-Exos influences this effect. Since ADMSC-Exos downregulated the expression of α-SMA in fibroblasts, Li C. et al. (2022) predicted that ADMSC-Exos increased the migration rate of fibroblasts from the periphery to the wound center, thereby reducing surface tension and inhibiting excessive formation of PS.

Exosomes can be used as a treatment to reduce the incidence of PS and avoid the immune risks associated with stem cell transplantation. Exosomes can also be stored for a long time and used at any time, which provides convenience for rapid clinical application. Most importantly, the vesicles are small enough to travel smoothly through the capillary circulation to the treatment site. However, the extraction rate of exosomes is very low, and they are easily cleared in the body. In order to develop a new way of exosome therapy, the extraction rate of exosomes should be further increased and the clearance rate of exosomes in the circulatory system should be reduced.

## 4 Discussion

In order to search for batter treatment options for PS, a variety of different approaches have been used clinically, such as surgery, medication, and radiation therapy. However, these treatments have certain side effects and are prone to relapse. The application of new technologies and new materials has opened up new ways to treat PS, such as MNs, PDT, novel photosensitizers and exosomes. Their application can reduce the side effects of treatment, avoid secondary trauma and improve curative effect. To enhance the therapeutic effect of PS, it is necessary to combine scar treatment drugs with new technologies and materials in a more reasonable manner. Additionally, efforts should be made to reduce the production cost of new materials, minimize their losses and side effects, and improve their physical properties and purity. In summary, it is hoped that new technologies and materials can closely correlate the generation and treatment of PS, to be widely developed and applied in the future.
